# Clinical Efficacy of Chinese Medicine in Treating Adult Henoch–Schönlein Purpura: A Meta‐Analysis

**DOI:** 10.1155/bmri/9725971

**Published:** 2026-07-03

**Authors:** Otgongerel Nergui, Jin Huan Wang, He Dan Di, Ding Ding Li, Yang Yang Li, Xianghui Wan, Wei Yi, Wang Bin, Saruultuya Nergui, Ganchimeg Dondov, Tegshjargal Badamjav, Tulgaa Lonjid, Batbold Batsaikhan

**Affiliations:** ^1^ Department of Hematology, First Affiliated hospital, Heilongjiang University of Chinese Medicine, Harbin, China, hljucm.edu.cn; ^2^ Department of Internal Medicine, Institute of Medical Sciences, Ulaanbaatar, Mongolia; ^3^ Department of Internal Medicine, Heilongjiang University of Chinese Medicine, Harbin, China, hljucm.edu.cn

**Keywords:** Chinese and Western medicine, Henoch–Schönlein purpura, traditional Chinese medicine

## Abstract

**Introduction:**

This meta‐analysis evaluates the efficacy of traditional Chinese medicine, alone or in combination with Western medicine, for the treatment of adult Henoch–Schönlein purpura.

**Methods:**

We searched clinical randomized controlled trials on the treatment of adult Henoch–Schönlein purpura with traditional Chinese medicine from the Chinese Journal Literature Database (CNKI), Wanfang Database, Embase, PubMed, Cochrane, Scopus, the Journal of Pharmacopuncture, and the National Library of China. After removing duplicate literature and reviewing titles, we selected the final studies based on our inclusion and exclusion criteria. These studies were assessed for bias using the Cochrane risk of bias assessment tool, and statistical analysis was performed using Review Manager 5.3.0 software. The PRISMA guidelines were followed when conducting the meta‐analysis.

**Results:**

Eighteen studies were included, with a combined sample size of 1632 cases, including 811 in the experimental group and 821 in the control group. The results indicated that traditional Chinese medicine, whether used alone or in combination with Western medicine, was more effective than Western medicine alone in terms of overall effectiveness and improvement in skin purpura, digestive tract, and joint symptoms. We found that the total clinical effective rate of patients in the experimental group was higher than that of the control group (OR = 6.04, 95% CI [4.01, 9.10], *p* < 0.001). Meta‐analysis indicates that the difference between the control and experimental groups in improving skin purpura symptoms is statistically significant (SMD = −1.09, 95% CI [−1.22, −0.96], *p* < 0.001). We found that a more significant improvement occurred in the experimental group than in the control group (SMD = −0.95, 95% CI [−1.08, −0.82], *p* < 0.001). People in the experimental group showed a greater decrease in joint symptoms than those in the control group (SMD = −0.95, 95% CI [−1.08, −0.82], *p* < 0.001).

**Conclusion:**

Traditional Chinese medicine, either alone or combined with Western medicine, is more effective than Western medicine alone in treating Henoch–Schönlein purpura. It also shows superior efficacy in improving skin purpura, digestive tract, and joint symptoms.

## 1. Introduction

Henoch–Schönlein purpura (HSP), also known as IgA vasculitis, is a systemic vasculitis syndrome characterized primarily by small‐vessel inflammation due to an abnormal deposition of immunoglobulin A (IgA) in the blood vessel walls. Its main clinical manifestations include nonthrombocytopenic skin purpura, gastrointestinal mucosal bleeding, joint swelling and pain or arthritis, and kidney injury (such as hematuria and/or proteinuria) [1–3]. The incidence of HSP has been increasing annually [3–5]. According to estimates, the average annual incidence of HSP in adults is 0.8–1.8 in 100,000 population. In children′s cases, the illness is often self‐limiting; in adults, it is more intricate, with renal failure manifesting in almost 50% of individuals exhibiting renal involvement. Gastrointestinal and kidney involvement are major causes of mortality in adults [4–8]. Additionally, the disease may present with rare manifestations, including neurological, pulmonary, ocular, circulatory, and reproductive system involvement, which can lead to misdiagnosis and delayed treatment [8, 9].

To address both the immediate symptoms and long‐term outcomes associated with HSP, a comprehensive approach is needed to optimize patient care and improve overall prognosis [10]. The management of HSP focuses primarily on supportive care, and the use of adrenal glucocorticoids is also effective in clinical practice [11]. Nevertheless, long‐term or repeated usage of glucocorticoids may result in adverse effects such as immunosuppression, metabolic disorders, and higher susceptibility to infections, indicating the need for alternate or complementary therapeutic approaches.

In China, Chinese herbal medicine is among the most commonly used treatments for HSP. TCM has demonstrated additional beneficial effects, including decreased blood coagulation, reduced proteinuria, and improved TCM symptoms [12]. HPS is considered a representative condition with a notably effective treatment using Chinese medicine alone, highlighting the strengths and unique aspects of TCM. Despite this, there is a lack of systematic reviews on the use of Chinese medicine for treating adult HSP. We aim to conduct a meta‐analysis of the existing literature on the clinical efficacy of traditional Chinese medicine in the treatment of Henoch–Schönlein purpura in adults.

## 2. Materials and Methods

### 2.1. Literature Searching Strategy

#### 2.1.1. Search Database

• Chinese National Knowledge Infrastructure (CNKI), Wanfang Database (Wanfang), Cochrane Central Register of Controlled Trials (CENTRAL), Embase, PubMed, Scopus, Journal of Pharmacopuncture, National Library of Medicine, and ScienceDirect.

#### 2.1.2. Keywords

• English: Allergic purpura, Henoch–Schönlein, HSP, vasculitis, Henoch‐purpura, Schönlein‐purpura, traditional Chinese medicine, Chinese medicine, Chinese herb, integrated Chinese and Western medicine, TCM.
• The keywords for literature searching are “Xijiao Dihuang Decoction–犀角地黄汤” and “Henoch‐Schönlein purpura.” “rhinoceros Dihuang decoction.”

#### 2.1.3. Searching Strategy

According to the characteristics of different databases, different methods are selected for systematic search, and the logical combination is as follows:

English:

1. “HENOCH‐SCHÖNLEIN PURPURA” OR “HENOCH PURPURA” OR “SCHÖNLEIN‐PURPURA” OR “HSP”
2. “ALLERGIC PURPURA” OR “AP”
3. “VASCULIT” OR “VASCULITIS”
4. “TRADITIONAL CHINESE MEDICINE” OR “CHINESE HERB” OR “CHINESE MEDICINE” OR “INTEGRATED CHINESE AND WESTERN MEDICINE” OR “TCM”
5. “RANDOMIZED CONTROLLED TRIAL” OR “RANDOMIZED” OR “RCT”
6. “RHINOCEROS DIHUANG DECOCTION”
7. “XIJIAO DIHUANG DECOCTION–犀角地黄”
8. #1 AND #2 AND #3 AND #4

### 2.2. Selection Criteria

#### 2.2.1. Inclusion Criteria

Only randomized controlled trials (RCTs) evaluating the use of TCM for the treatment of HSP will be considered, and studies must be published in Chinese or English. Participants must be aged 18 years or older, meet clear diagnostic criteria for HSP, and be of any gender, race, or disease duration. The intervention must involve TCM, specifically the rhinoceros Dihuang decoction, either alone or in combination with Western medicine, alongside general treatment measures that address emotional well‐being, diet, daily rest, and allergen avoidance. The control group should receive conventional Western medicine for HSP, with no specific dosage requirements for TCM. Studies must report on the following outcome indicators: total clinical effectiveness rate, improvement in skin purpura symptoms, improvement in gastrointestinal symptoms, and improvement in joint symptoms.

#### 2.2.2. Exclusion Criteria

Exclusion criteria include the following:

• Diagnostic criteria: Literature with inconsistent or unclear Western medical diagnostic criteria for HSP will be excluded.
• Study type: Case reports, experience summaries, animal studies, and other nonresearch literature, such as opinion pieces or theoretical reviews, will be excluded.
• Outcome measures: Studies where the reported outcome indicators do not meet the specified inclusion criteria will be excluded.
• Comorbidity: Studies involving patients with other serious or significant diseases (malignancies, autoimmune diseases, heart failure, and osteoarthritis) that may confound the results will be excluded.
• Data quality: Studies with incomplete or unreliable research data that cannot be adequately analyzed will be excluded.
• Baseline characteristics: Studies with significant imbalances in baseline characteristics between intervention and control groups will be excluded.
• Duplicate publications: Only one study with complete data will be retained for each set of repeated publications to avoid redundancy.

### 2.3. Data Extraction

**Figure 1:**
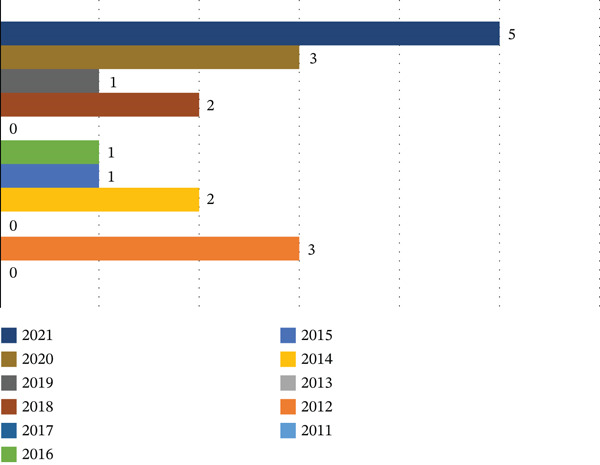
The number of publications by year in our screening.

In Figure 1, we displayed the total number of publications since 2011 by year in our screening. Between 2020 and 2021, the majority of the included studies were published.

#### 2.3.1. Literature Screening Process

1. Literature import and duplicate removal: All identified literature is imported into Zotero, a reference management software where initial duplicate records are systematically removed to ensure that each study is represented only once.
2. Preliminary screening: The remaining literature is evaluated by two independent reviewers based on their titles and abstracts, filtering out studies that do not meet the basic inclusion criteria.
3. Full‐text review: The full texts of literature that pass the preliminary screening are thoroughly reviewed. Each study is assessed according to the predefined inclusion and exclusion criteria to determine its suitability for the meta‐analysis.
4. Resolution of disagreements: If disagreements arise between the two reviewers during the screening process, they will attempt to reach a consensus through discussion. If a consensus cannot be reached, a third reviewer will be consulted to provide an impartial resolution.

#### 2.3.2. Methodological Quality Evaluation

The quality of the included studies was assessed using a primary tool: the Cochrane Collaboration′s risk of bias tool.

This assessment was carried out by two professional evaluators, with any disagreements resolved through negotiation or third‐party adjudication.

Risk of bias assessment using the Cochrane tool:

o. Random sequence generation: evaluation of the method used to generate random sequences in the studies.
o. Allocation concealment: assessment of whether the allocation of participants to intervention or control groups was concealed.
o. Blinding of participants and personnel: determination of whether researchers and participants were blinded to the intervention.
o. Blinding of outcome assessors: Examination of whether those assessing the outcomes were blinded to group assignments.
o. Incomplete outcome data: analysis of the completeness of outcome data and handling of dropouts or missing data.
o. Selective reporting: evaluation of whether the study selectively reported outcomes based on the results.
o. Other sources of bias: consideration of any other potential sources of bias not covered by the above categories.

The risk of bias was categorized as low, high, or unclear. Results were input into the Review Manager 5.3.0 software to create a risk of bias assessment chart.

A score of 3 or less indicated low quality, whereas a score greater than 3 indicated high quality. Evaluation results were determined by two professional evaluators, with any disagreements resolved through discussion or a third‐party ruling.

The relevant data from the included studies were extracted by two independent researchers and compiled into Excel tables for analysis. To ensure the accuracy and completeness of the data, the researchers exchanged and verified their data extractions. The following data elements were systematically extracted:

1. General information:
  o. Publication year: the year in which the study was first published.
  o. Diagnostic criteria: the criteria used for diagnosing HSP in the study.
  o. Study design: type of study and file types.
2. Subjects
  o. Sample size: total number of participants in each study.
  o. Randomization method: description of the method used for randomizing participants.
  o. Demographics: gender distribution, age range, and disease duration of the participants.
  o. Baseline characteristics: initial health status and other relevant baseline data of the participants.
3. Intervention measures:
  o. Treatment methods: specific treatments were administered to the treatment group and the control group.
  o. Drug information: names of drugs used, dosage forms, and dosage regimens.
  o. Composition and duration: details about the composition of the treatment and the duration of the treatment course.
4. Outcome indicators:
  o. Total clinical effective rate: the overall rate of clinical effectiveness reported in the studies.
  o. Skin purpura: improvement in skin purpura symptoms.
  o. Gastrointestinal symptoms: improvement in digestive tract symptoms.
  o. Joint symptoms: improvement in joint symptoms.

### 2.4. Statistical Analysis

The statistical software RevMan 5.3.0, developed by the Cochrane Collaboration, was used to conduct the meta‐analysis of the outcome indicators. The analysis followed the following steps:

1. Publication bias analysis:
  o. Publication bias was assessed using a funnel plot and the Egger test when there were ≥ 10 studies.
  o. The funnel plot, created using RevMan 5.3.0, plots effect sizes on the horizontal axis and sample sizes on the vertical axis. A symmetrical distribution of points around the centerline suggests minimal bias.
  o. The Egger test statistically evaluated the funnel plot asymmetry. A *p* value of > 0.05 indicates no significant publication bias.
2. Data type determination:
  o. Odds ratio (OR): used for dichotomous outcomes.
  o. Standardized mean difference (SMD): applied for continuous measurement data.
  o. Each effect size was reported with a 95% confidence interval (CI).
3. Heterogeneity assessment:
  o. Heterogeneity among studies was evaluated using the I^2^ statistic.
  o. If I^2^ ≤ 50%, indicating low heterogeneity, a fixed‐effect model was used.
  o. If I^2^>50%, indicating substantial heterogeneity, a random‐effects model was employed. Further investigation into the source of heterogeneity was performed through subgroup or sensitivity analyses.
  o. If identifiable heterogeneity factors were excluded, the fixed‐effect model was reapplied. If the sources of heterogeneity could not be identified, a descriptive analysis based on the original literature was conducted.
4. Forest plot:
  o. A forest plot was generated with RevMan 5.3.0. Each point represents an effect size from a study, with each horizontal line indicating the 95% CI of that effect size. The length of the horizontal line is inversely proportional to the sample size.
  o. The diamond shape in the forest plot represents the combined 95% CI. If the diamond intersects the vertical line of no effect, the result is not statistically significant.
5. Funnel plot analysis:
  o. The funnel plot was used to visually inspect publication bias. The horizontal axis represents effect sizes, and the vertical axis represents sample sizes. The center vertical line indicates the combined OR value, with skew lines representing the 95% CI.
  o. A symmetrical distribution of dots around the centerline in an inverted funnel shape indicates no significant bias, whereas asymmetry suggests potential publication bias.

By employing these statistical methods, the meta‐analysis is aimed at ensuring a rigorous and accurate assessment of the included studies, which address both efficacy and potential biases in the data.

The study was conducted in accordance with the Declaration of Helsinki and approved by the Institutional Review Board of the Institute of Medical Sciences, Mongolia (Protocol Code 23/01; date of approval is March 27, 2023) and ethical approval from the Institutional Ethics Committee of the Institute of Medical Sciences, Mongolia No. 07).

## 3. Results

### 3.1. Identification of Relevant Studies

**Figure 2:**
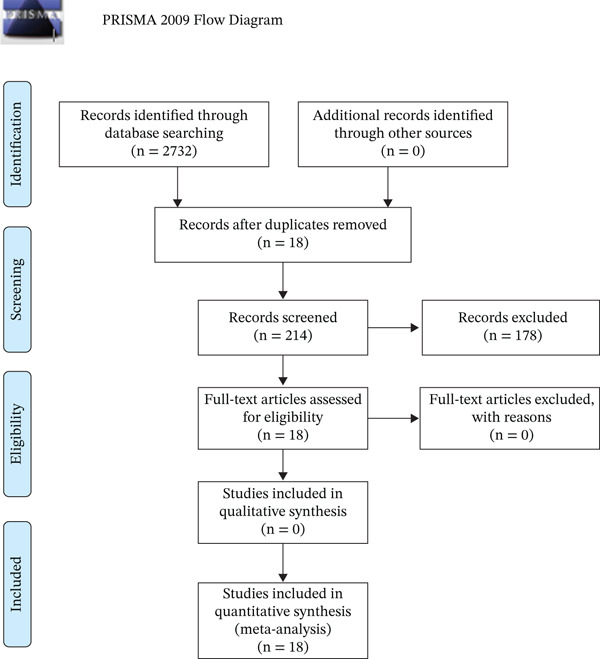
Literature screening flow chart.

Following the search strategy established for this study, we systematically searched nine databases: Embase, PubMed, Cochrane, Scopus, the Journal of Pharmacopuncture, the National Library of China, CNKI, the Wanfang Database, and ScienceDirect. The initial search yielded 214 relevant articles. We identified 18 duplicate entries using Zotero literature management software, and we further reviewed 196 articles. After screening titles and abstracts, we excluded 102 articles that met our exclusion criteria. From these, 58 articles included the cases of pediatric HSP, and 18 were nonclinical studies. Therefore, we finally included 18 articles for full‐text review (Table 1). Upon detailed examination, we found that some articles either lacked full text or contained incomplete data, rendering them unsuitable for extracting outcome indicators. Consequently, the final selection process for the 18 included studies is illustrated in Figure 2 (literature screening flow chart).

**Table 1:** Basic characteristics of the included studies.

Authors	Medicine	Control group	Jadad score	Age (case/control)	Disease year
Hu N, et al. (2021) [13]	Chinese medicine	Western	2	70.2±9.37/73±7.6	3.6±0.8/4.2±1.0
Yu J, et al. (2020) [14]	Western	Chinese medicine	2	51.8±11.1/47.6±	26.3±7.3/25.6±3.6
Li WX, et al. (2021) [15]	Western	Chinese medicine	2	48.2±11.3/47.3±	5.2±2.3/6.2±1.8
S. XIN, et al. (2020) [16]	Western	Chinese medicine	2	70.6±9.6/72.1±5.	15.5±2.6/14.8±1.8
Kong Z, et al. (2021) [17]	Chinese medicine	Western	2	63±3.21/67.2±2.3	26.5±1.8/25.8±3.6
Suyeon Cho, et al. (2021) [18]	Chinese medicine	Western	2	65.37±8.5/63.3±	14.5±0.5/16.2±0.9
Wang Jun Ji, et al. (2012) [19]	Chinese medicine	Western	2	23.8±10.8/19.3±	15.6±0.3/14.8±2.3
Xua Ning, et al. 2014 [20]	Chinese medicine	Western	2	62.3±6.9/65.2±7.	19.7±0.3/18.2±1.2
Yu Gui Gui, et al. (2012) [21]	Chinese medicine	Western	2	32±9.6/33.2±3.6	18.2±1.2/17.2±3.3
Shi Xiao Men, et al. (2021) [22]	Chinese+Western	Western	2	54±9.8/56.2±7.8	7.2±0.8/6.8±1.3
Jiang M, et al. (2019) [23]	Chinese+Western	Western	2	56.3±6.3/58.2±3.	6.3±0.1/5.9±8.9
Ren P, et al. (2012) [24]	Western	Chinese medicine	2	68.2±1.2/71.5±3.	14.7±0.8/13.5±9.7
Huang X, et al. (2018) [25]	Western	Chinese medicine	2	49.6±9.7/51.2±9.	13.8±1.2/12.8±0.9
Huang Chuang, et al. (2016) [26]	Chinese+Western	Western	2	72.3±9.4/75.2±3.	8.9±0.2/7.8±3.2
Audemard‐Verger A, et al. (2015) [3]	Western	Chinese medicine	2	52.1±8.6/55.6±1.	6.2±1.2/5.7±3.2
Calvo‐Río V, et al. (2014) [27]	Western	Chinese medicine	2	47.2±6.9/71.2±7.	8.1±2.3/7.9±0.8
Gazel U, et al. (2020) [28]	Western	Chinese medicine	2	62.1±5.8/65.2±1.	4.5±0.2/3.9±3.6
Hu Yan et al. (2021)[29]	Chinese+Western	Western	3	56±8.7/61.2±9.8	7.00±2.7/8.00±2.4

### 3.2. Methodological Quality

**Figure 3:**
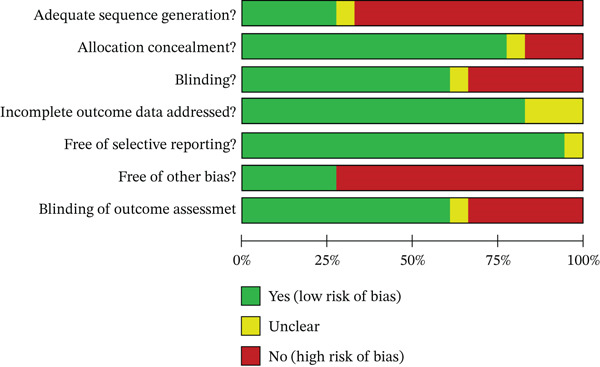
Bias risk bar chart.

The 18 included studies were evaluated using the Cochrane Risk of Bias tool [13–30]. In Figure 3, the bias risk bar chart provides a detailed assessment of the bias risk for the included studies.

• Selection bias: All studies reported on the randomness of assignment. Among these, five studies [16, 20, 21, 23, 27] employed the random number table method, which was associated with a low risk of bias. Regarding the other studies that did not specify the randomization method, the risk of bias was deemed unclear.
• Allocation concealment: Two studies [13, 28] employed opaque envelopes for allocation concealment, resulting in a low risk of bias. The other studies did not specify their methods of allocation concealment, leading to an unknown risk of bias.
• Implementation bias: None of the studies reported using double blinding or other blinding methods during implementation, resulting in a high risk of bias.
• Measurement bias: The blinding of outcome assessors was not reported in the studies; thus, the risk of measurement bias remains unclear.
• Follow‐up bias: All studies provided complete outcome data, indicating a low risk of bias in follow‐up.
• Reporting bias: No selective reporting of study outcomes was observed, and the risk of reporting bias was considered low across all indicators.
• Other bias: All studies were compared against baseline data, with the risk of other biases being low.

Two studies focused on patients aged 14 years [20], and one study included patients aged 16 years [21].

### 3.3. Total Clinical Efficacy

**Figure 4:**
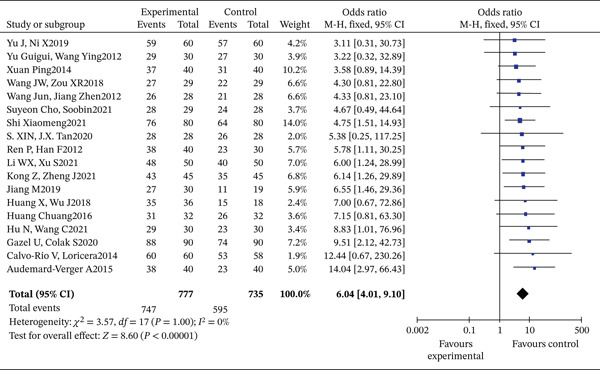
Forest map of clinical total effective rate.

There were 18 studies, including 1632 cases; 811 were in the experimental group and 821 in the control group. The heterogeneity test showed no significant heterogeneity among the 21 studies (*p* > 0.05, I^2^ = 0*%*), so a fixed‐effect model was employed. As shown in Figure 4, the total clinical effective rate in the experimental group was higher than that in the control group (OR = 6.04, 95% CI [4.01, 9.10], *p* < 0.001).

### 3.4. The Improvement of Skin Purpura Symptoms

**Figure 5:**
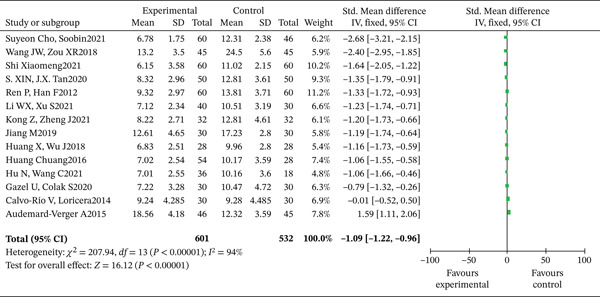
Improvement of skin purpura symptoms after treatment forest map.

A total of 14 studies, including 601 people (control group: 532; experimental group: 601), evaluated the improvement in skin purpura symptoms. The heterogeneity test indicated heterogeneity among the included studies (*p* < 0.001, I^2^ = 94*%*), so a random‐effects model was used. Meta‐analysis indicated that the difference between the control and experimental groups in improving skin purpura symptoms is statistically significant (SMD = −1.09, 95 CI [−1.22, −0.96], *p* < 0.001), as shown in Figure 5.

### 3.5. The Improvement of Gastrointestinal Symptoms

**Figure 6:**
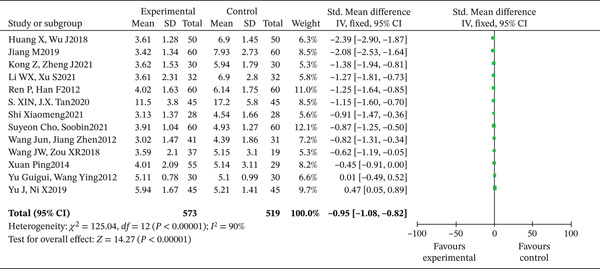
Forest map of improvement of gastrointestinal symptoms after treatment.

Of the 18 included studies, 13 evaluated the change in digestive tract symptoms, involving a total of 1092 people, comprising 519 in the control group and 573 in the experimental group. The heterogeneity test indicated considerable heterogeneity among these studies (*p* < 0.001, I^2^ = 90*%*). Therefore, we employed a random‐effects model for the analysis. We found that a more significant improvement occurred in the experimental group than in the control group (SMD = −0.95, 95% CI [−1.08, −0.82], *p* < 0.001), as shown in Figure 6.

### 3.6. The Improvement of Joint Symptoms

**Figure 7:**
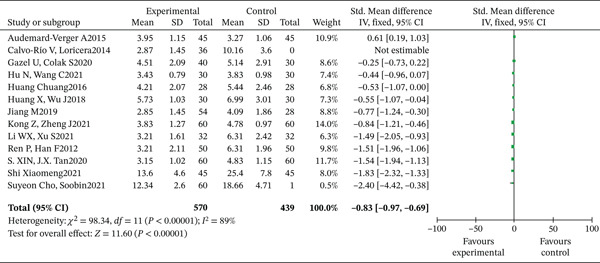
Forest map of improvement of joint symptoms after treatment.

A total of 13 studies reported improvements in joint symptoms. These studies included 1009 people: 439 in the control group and 570 in the experimental group. According to the heterogeneity test, we found substantial heterogeneity among these studies (*p* < 0.001, I^2^ = 89*%*). Consequently, a SMD and a random‐effects model were applied. In Figure 7, people in the experimental group showed a greater decrease in joint symptoms than those in the control group (SMD = −0.83, 95% CI [−0.97, −0.69], *p* < 0.001).

### 3.7. Detection of Publication Bias

**Figure 8:**
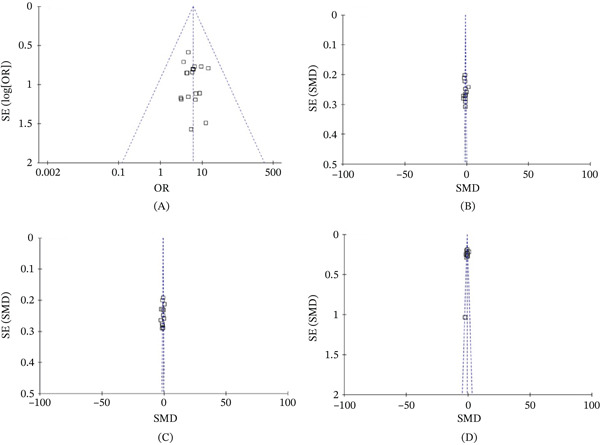
The Funnel plots of the effective rate of TCM.

Figure 8 reports the funnel plots of the effective rate of TCM (A), the total effective rate after treatment (B), the improvement in skin purpura symptoms after treatment (C), the improvement in gastrointestinal symptoms after treatment (D), and the improvement in joint symptoms after treatment. Each plot depicts symmetry between the left and right sides, indicating low publication bias.

## 4. Discussion

HPS is a systemic vasculitis that predominantly affects small blood vessels and presents with symptoms such as nonthrombocytopenic skin purpura, gastrointestinal mucosal bleeding, joint swelling or pain, and renal involvement. The incidence is notably higher in children compared with adults, with varying severity and recurrence rates. TCM has been extensively researched for pediatric cases, but studies on adult HSP are limited, despite its clinical significance and tendency to recur in adults.

Our meta‐analysis of 18 RCTs [3, 13–29] highlights several key findings regarding the efficacy of TCM in treating adult HSP. The results demonstrate that TCM—whether administered alone or in combination with Western medicine—shows a significant advantage over Western medicine alone in improving overall clinical effectiveness. The fixed‐effects model yielded an OR (OR = 6.04, 95% CI [4.01, 9.10], *p* < 0.001), indicating a robust benefit of TCM treatments.

Specifically, TCM or combined treatments were significantly more effective in alleviating skin purpura symptoms than Western medicine (SMD = −1.09, 95% CI [−1.22, −0.96], *p* < 0.001). The high heterogeneity among studies underscores the variability in treatment responses, which might be due to differences in TCM formulations, patient characteristics, or study methodologies.

In managing gastrointestinal symptoms, TCM or combined therapies demonstrated superiority over Western medicine alone (SMD = −0.95, 95% CI [−1.08, −0.82], *p* < 0.001). Similarly, TCM was more effective in controlling joint symptoms (SMD = −0.83, 95% CI [−0.97, −0.69], *p* < 0.001). These findings align with the traditional use of TCM in targeting systemic inflammation and improving overall symptom management. Nevertheless, a systematic assessment of the adverse consequences of TMC has not yet been conducted.

Publication bias assessments revealed symmetrical funnel plots (Figures 6, and 8), suggesting minimal risk of bias in the included studies. However, limitations such as small sample sizes, inconsistent safety reporting, and variability in TCM prescriptions could affect the reliability of the results. Most studies did not comprehensively report adverse events, making it difficult to assess the full safety profile of TCM treatments. Additionally, the lack of standardized treatment protocols across studies introduces variability that may influence the outcomes. Moreover, the clinical heterogeneity of TCM interventions may limit generalizability.

Our analysis highlights the efficacy of TCM in the treatment of adult HSP, particularly in improving outcomes for purpura, gastrointestinal, and joint symptoms. TCM, whether administered alone or in conjunction with Western medicine, is more effective than Western medicine alone. This approach may lead to improved patient outcomes and reduced recurrence rates.

Despite these promising findings, further research is essential. Future studies should involve standardized treatment regimens and thorough safety evaluations to confirm these results and refine treatment protocols. The integration of TCM with Western medicine represents a potentially effective strategy for managing this complex condition, offering a more comprehensive approach to treatment and patient care.

In conclusion, our analysis has shown that treatment with a combination of TCM and Western medicine is more effective in improving skin purpura, digestive tract, and joint symptoms.

**Nomenclature**
C3: Complement 3
CNKI: China National Knowledge Infrastructure
Embase: Excerpta Medica Database
Fixed: Fixed‐effect methods
HSP: Henoch–Schönlein purpura
HSPN: Henoch–Schönlein purpura nephritis
IgA: Immunoglobulin A
IgAV: Immunoglobulin A vasculitis
IgG: Immunoglobulin G
IV: Inverse variance
OR: Odds ratio
Pub: Pubmed
RCT: Randomized controlled trial
RIB: Risk‐of‐bias
TCM: Traditional Chinese medicine
VC: Vitamin C

## Funding

No funding was received for this manuscript.

## Ethics Statement

The study was conducted in accordance with the Declaration of Helsinki and approved by the Institutional Review Board of the Institute of Medical Sciences, Mongolia (Protocol Code 23/01; date of approval is March 27, 2023), and ethical approval from the Institutional Ethics Committee of the Institute of Medical Sciences, Mongolia (No. 07).

## Conflicts of Interest

The authors declare no conflicts of interest.

## Data Availability

Data sharing is not applicable to this article as no datasets were generated or analyzed during the current study.
